# Machine-learning-based Phenomapping of Patients with Keratinocyte Carcinoma: Data-driven Subgrouping by Disease Burden, Comorbidities and Socioeconomic Status

**DOI:** 10.2340/actadv.v106.adv-2026-0843

**Published:** 2026-08-13

**Authors:** Johan Sieborg, Emily Wenande, Mia-Louise Nielsen, Mads Nielsen, David Thein, Merete Haedersdal

**Affiliations:** 1 Department of Dermatology, Copenhagen University Hospital – Bispebjerg and Frederiksberg, Copenhagen, Denmark; 2 Pioneer Centre for AI, University of Copenhagen, Copenhagen, Denmark; 3 Department of Clinical Medicine, Faculty of Health and Medical Sciences, University of Copenhagen, Copenhagen, Denmark

**Keywords:** Basal cell carcinoma, Squamous cell carcinoma, Bowen disease, Epidemiology, Keratinocyte carcinoma, Non-melanoma skin cancer, Machine learning

## Abstract

Keratinocyte carcinoma (KC) places a considerable and growing burden on healthcare systems. Given the KC population’s heterogeneity, tailored clinical pathways are needed to accommodate diverse management needs. This study applied machine learning (ML)-based phenomapping to identify distinct real-world subgroups within a national KC population using demographic and medical history variables. The study included KC patients treated in publicly-funded, office-based dermatology practices and registered in the Danish Skin Cancer Registry (2014–2022). In 106,490 KC patients, 7 ML-derived clusters were identified. One cluster (22.2%; *n*=23,590) consisted primarily of young, well-educated high-income medically noncomplex females presenting with low-risk basal cell carcinomas (BCCs). Four clusters (55.2%; *n*=58,736) comprised patients with a greater dermatological disease burden, characterized by facial BCCs, multiple BCCs, a greater proportion of squamous cell carcinomas (SCCs), a history of skin cancer or previous actinic keratosis-related treatment. The remaining 2 clusters (22.7%; *n*=24,164) comprised highly comorbid patients with a greater proportion of SCCs, a high number of KCs on the head and neck and high immunosuppressive drug exposure. ML-derived phenomapping of a national KC population identified distinct, clinically relevant KC subgroups, providing a basis for tailored clinical pathways with differentiated resource needs.

**SIGNIFICANCE**

The large and growing keratinocyte carcinoma (KC) population comprises several patient subgroups with different disease characteristics, demographic backgrounds, comorbidities and critically, management needs. In 106,490 KC patients, 7 machine learning-derived subgroups were identified. One group (22.2% of the patients) was characterized by younger patients with low-risk tumours and few comorbidities. Most patients (55.2%) belonged to 4 individual groups who expressed high KC disease burden and skin-related comorbidities. The remaining 2 groups (22.7%) included patients with relatively many high-risk KCs and many comorbidities. Our findings are important for advancing personalized patient management and optimizing resource allocation to improve KC care.

Keratinocyte carcinoma (KC) is the most common form of skin cancer worldwide, and its incidence has steadily increased over the past decade (1–3). Additionally, the incidence rates are projected to rise 140% by 2050 among individuals older than 65 years (4). The KC patient population is highly heterogenous, encompassing individuals on immunosuppressive therapy (5, 6); those with sun-damaged skin (7); genetic susceptibilities (8); previous skin cancer history (9, 10) and wide variation in age, health status (11, 12) and socioeconomic status (SES) (13, 14). Patient-centric segmentation of the KC population can better define distinct patient phenotypes, their risk profiles and care needs, supporting more efficient resource allocation.

A patient-centric approach integrated across the entire KC care pathway may accommodate individuals with medical complexity due to extensive comorbidities (15) and account for the broader heterogeneity of the KC population. Furthermore, socioeconomic and demographic factors may considerably shape the individual KC burden, care utilization (16) and ability to attend regular follow-up. In general, there is no “one-size-fits-all” therapy or monitoring strategy, and modern approaches are shifting towards broader patient-centric stratification, incorporating demographics, comorbidities and socioeconomic determinants at a population level to guide appropriate care setting and therapies (17–19). Phenomapping involves applying machine learning (ML) or statistical clustering methods to more objectively identify distinct patient subgroups. Unsupervised ML methods for clustering of patients are increasingly used in medical research. Previous studies have applied clustering algorithms to patients with diabetes (20), hypernatremia (21) and psoriasis (22) to find distinct patient phenotypes and evaluate clinical outcomes. In emergency departments where rapid and efficient triaging is essential, phenomapping has been used to identify clinically relevant patient subgroups (23). These methods are useful for developing personalized patient management, as patients may benefit from specialized care tailored to their characteristics and medical history. This is particularly feasible in Denmark, where national registries contain comprehensive information on each individual, with high completeness over several decades (24). Thus, this study aimed to apply phenomapping to assess the KC population to identify distinct patient subgroups using ML clustering with the potential to uncover previously unrecognized multidimensional patient profiles.

## METHODS

### Data sources and study population

Source data were extracted from the Danish Skin Cancer Registry (*Hudkræftdatabasen*), which contains records of patients treated for KC in office-based dermatological practices participating in the state-funded public healthcare system across Denmark. This national registry is part of the Danish Clinical Quality Program for National Clinical Registries and includes data on treatment choice and tumour characteristics such as basal cell carcinoma (BCC) histological subtype, lateral tumour size, anatomical location and recurrent tumours. Demographic data including patient sex, age, individual annual income, education level, as well as medication and other comorbidities were extracted from the Civil Registration System, the Income Statistics Register, the Danish Education Register, the Danish National Prescription Registry and the Danish National Patient Register (24).

The study population comprised all KC patients with either a nodular BCC (nBCC), superficial BCC (sBCC), squamous cell carcinoma (SCC) or Bowen disease (BD) diagnoses treated between 2014 and 2022 with records in the Danish Skin Cancer Registry. Each KC patient was only included once, with baseline data collected at first recorded treatment.

### Study objective and variable definitions

The study’s objective was to apply phenomapping in KC patients to identify distinct KC patient subgroups based on healthcare utilization, medical history and patient characteristics using unsupervised ML clustering.

Healthcare utilization included the number of hospital-registered diagnoses and number of procedures in dermatological departments. Hospital-registered diagnoses were counted as all International Classification of Diseases 10 (ICD-10) codes recorded in a hospital setting prior to KC treatment. Dermatological procedures were identified using the Danish healthcare classification system (Sundhedsvæsenets Klassifikationssystem, SKS) codes KQ* or KYQ*, performed at dermatological departments, counting each unique SKS code at most once per day.

The medical history was derived from a broad range of hospital diagnoses, hospital procedures and prescribed medications. Previous skin cancer was defined as any nonmelanoma skin cancer (C44*) or melanoma skin cancer (C43*) ICD-10 diagnosis, counting at most 1 per day. Number of tumours at consultation was defined as the number of KC tumours recorded at baseline in the Danish Skin Cancer Registry. Hospital-registered (ICD-10) actinic keratosis (AK; L570*) were recorded prior to inclusion. Medication use was determined from filled prescriptions before inclusion and included immunosuppressive drugs (SKS: ML04* and BOHJ* (excluding antibody treatments) and Anatomical Therapeutic Chemical Classification System (ATC) codes L04*), in addition to prednisolone, ciclosporin, azathioprine, methotrexate, imiquimod, fluorouracil cream and photodynamic therapy (PDT) (see Supplementary Material 1 for specific codes). A modified Charlson Comorbidity Index (CCI) was calculated from ICD-10 diagnoses, excluding age and skin cancer to avoid collinearity. Smoking history was determined through a combination of hospital diagnoses (ICD-10) and medication ATC codes described in detail elsewhere (25). History of alcohol abuse was determined using a combination of hospital diagnosis of alcohol abuse (or related conditions such as alcoholic liver disease) and medications use for alcohol dependence, or participation in treatment interventions for alcohol dependence.

Demographic information included sex, age, annual income and education level. Education was categorized as higher education (bachelor or higher educations), basic education (elementary-school or high-school), vocational education or unknown. Income was the average annual income 3 years prior to inclusion.

KC tumour characteristics were analysed for each cluster but not used in the clustering process. Anatomical location was recorded by dermatologists using ICD-10, grouped into unspecified face (C443, D043), lip (C440, D040), eye (C441, D041), ear (C442, D042), neck or scalp (C444, D044), upper extremity (C446, D046), trunk (C445, D045) and lower extremity (C447, D047). Head and neck combined face, lip, eye, ear and neck or scalp, and body combined lower extremity, upper extremity and trunk. Lateral tumour size was grouped as <5 mm, 5–10 mm, 10–15 mm, >15 mm or unknown. Index date was defined as the date of the first recorded visit in the Danish Skin Cancer Registry.

### Statistics

Clusters were determined based on number of tumours at baseline, number of previous skin cancer tumours, number of procedures in dermatological departments, number of unique usages of imiquimod, fluorouracil or PDT (ranging from 0 to 3), smoke- or alcohol-abuse, CCI, number of hospital-registered diagnoses, education level, sex, age and annual income. All variables are based on information up to and including the time of diagnosis, with no later information included. These variables were selected based on clinical and patient relevance, guided by domain expertise. Missing numerical values were imputed with the median and categorical values with the most frequent category. Annual income was log-transformed to reduce the influence of outliers, and all numerical variables were standardized. Extreme outliers in annual income and number of hospital diagnoses were excluded. Variable correlations were assessed using Spearman rank test to avoid strong collinearity; the highest coefficient was –0.46 (between annual income and age). The optimal number of clusters was determined using a combination of the elbow method, the silhouette scores and cluster stability (see Materials S1). Clustering stability was assessed using bootstrapping (20 iterations), with the mean adjusted Rand Index score calculated for each cluster. Clustering was performed using the KPrototypes algorithm in Python, combining KMeans for numerical and KModes for categorical data. Heatmaps display percentage point differences between each cluster and all remaining clusters. All analyses were conducted in Python 3.7.4.

## RESULTS

### Population characteristics

The study included 106,490 unique KC patients with a median age of 72.5 years (interquartile range [IQR]: 63.5–79.4 years) and slightly more females (52.2%) than males (Table I). A larger portion of individuals were vocationally educated (39.0%), followed by groups with higher education (31.3%) and basic education (28.1%). The majority of KCs registered in the population were BCCs, accounting for 88.1% of all tumours at the index visit, while a smaller amount was SCC (7.3%) and BD (4.6%) (Table II). Most tumours were less than 10 mm (67.6%). Tumour location was relatively evenly distributed on the head and neck region (53.4%) or the body (46.6%). The number of KCs referred to other medical specialties was 23,364 (18.7%), while the remaining 81.3% were treated at an office-based dermatological clinic. Prior to the index date, 3.3% had a history of melanoma and 14.9% had a history of nonmelanoma skin cancer (Table III). Immunosuppressive therapy was noted in 5.6% of the population, and the comorbidity burden – measured by a modified CCI – averaged 0.7. A history of smoking or alcohol abuse was recorded in 14.2% and 4.0% of patients, respectively.

**Table I.** Patient characteristics

	Cluster 1	Cluster 2	Cluster 3	Cluster 4	Cluster 5	Cluster 6	Cluster 7	Overall
**Total number of patients**	23,590 (22.2%)	39,740 (37.3%)	10,518 (9.9%)	8,041 (7.6%)	437 (0.4%)	20,115 (18.9%)	4,049 (3.8%)	106,490 (100%)
**Sex, n(%**)								
Male	9,158 (38.8)	23,301 (58.6)	5,705 (54.2)	4,166 (51.8)	227 (51.9)	5,891 (29.3)	2,507 (61.9)	50,955 (47.8)
Female	14,432 (61.2)	16,439 (41.4)	4,813 (45.8)	3,875 (48.2)	210 (48.1)	14,224 (70.7)	1,542 (38.1)	55,535 (52.2)
**Age at treatment, median (IQR**)	55.3 (48.4–62.2)	74.5 (69.3–80.0)	74.2 (67.8–80.5)	75.3 (68.5–81.4)	76.7 (71.1–83.0)	77.0 (71.1–83.1)	78.5 (72.4–84.2)	72.5 (63.5–79.4)
**Annual income [DKK], median (IQR**)	805,390 (575,224–1,090,966)	332,817 (244,826–455,461)	354,784 (255,330–520,367)	386,625 (273,621–582,186)	333,590 (254,732–519,678)	314,231 (233,704–436,668)	316,391 (232,671–441,127)	387,567 (266,729–605,489)
**Number of hospital visits with diagnosis, median (IQR**)	12 (7–21)	13 (8–20)	16 (8–27)	21 (12–35)	37 (23–58)	41 (32–54)	41 (27–58)	18 (9–31)
**Highest achieved education, *n*(%**)								
Higher education	13,126 (55.6)	9,142 (23.0)	2,858 (27.2)	2,679 (33.3)	127 (29.1)	4,500 (22.4)	901 (22.3)	33,333 (31.3)
Basic education	3,456 (14.7)	10,569 (26.6)	2,998 (28.5)	2,117 (26.3)	120 (27.5)	9,255 (46.0)	1,371 (33.9)	29,886 (28.1)
Vocational education	6,855 (29.1)	19,273 (48.5)	4,460 (42.4)	3,095 (38.5)	179 (41.0)	5,985 (29.8)	1,692 (41.8)	41,539 (39.0)
Unknown education	153 (0.6)	756 (1.9)	202 (1.9)	150 (1.9)	11 (2.5)	375 (1.9)	85 (2.1)	1,732 (1.6)

**Table II.** Tumour characteristics

	Cluster 1	Cluster 2	Cluster 3	Cluster 4	Cluster 5	Cluster 6	Cluster 7	Overall
Total number of tumours	24,863 (19.9%)	39,740 (31.9%)	24,811 (19.9%)	9,358 (7.5%)	624 (0.5%)	21,017 (16.9%)	4,313 (3.5%)	124,726 (100%)
**Tumour diagnoses, *n*(%**)								
Nodular basal cell carcinoma	18,384 (73.9)	28,708 (72.2)	16,531 (66.6)	5,738 (61.3)	387 (62.0)	14,706 (70.0)	2,859 (66.3)	87,313 (70.0)
Superficial basal cell carcinoma	5,272 (21.2)	5,751 (14.5)	6,188 (24.9)	1,864 (19.9)	107 (17.1)	2,851 (13.6)	514 (11.9)	22,547 (18.1)
Bowen’s disease	528 (2.1)	1,813 (4.6)	1,149 (4.6)	560 (6.0)	38 (6.1)	1,332 (6.3)	293 (6.8)	5,713 (4.6)
Squamous cell carcinoma	679 (2.7)	3,468 (8.7)	943 (3.8)	1,196 (12.8)	92 (14.7)	2,128 (10.1)	647 (15.0)	9,153 (7.3)
Recurrent tumour	699 (2.8)	1,447 (3.6)	554 (2.2)	437 (4.7)	7 (1.1)	564 (2.7)	105 (2.4)	3,813 (3.1)
Number of tumours at treatment, median (IQR)	1 (1–1)	1 (1–1)	2 (2–3)	1 (1–1)	1 (1–2)	1 (1–1)	1 (1–1)	1 (1–1)
**Tumour size, *n*(%**)								
<5 mm	8,227 (33.1)	9,606 (24.2)	6,643 (26.8)	2,715 (29.0)	174 (27.9)	5,259 (25.0)	948 (22.0)	33,572 (26.9)
5–10 mm	10,165 (40.9)	16,302 (41.0)	10,034 (40.4)	3,683 (39.4)	242 (38.8)	8,680 (41.3)	1,690 (39.2)	50,796 (40.7)
10–15 mm	2,679 (10.8)	5,672 (14.3)	3,201 (12.9)	998 (10.7)	77 (12.3)	2,849 (13.6)	641 (14.9)	16,117 (12.9)
>15 mm	1,229 (4.9)	3,785 (9.5)	2,226 (9.0)	616 (6.6)	47 (7.5)	1,815 (8.6)	504 (11.7)	10,222 (8.2)
Unknown/irregular	2,546 (10.2)	4,331 (10.9)	2,684 (10.8)	1,334 (14.3)	84 (13.5)	2,405 (11.4)	526 (12.2)	13,910 (11.2)
**Anatomical locations, n(%**)								
**Head and neck (Face, ear, scalp or neck, lip, eye combined**)	11,298 (45.4)	24,461 (61.6)	9,968 (40.2)	5,004 (53.5)	284 (45.5)	12,866 (61.2)	2,738 (63.5)	66,619 (53.4)
Face	8,857 (35.6)	18,130 (45.6)	7,258 (29.3)	3,512 (37.5)	191 (30.6)	9,848 (46.9)	1,931 (44.8)	49,727 (39.9)
Ear	409 (1.6)	1,836 (4.6)	489 (2.0)	358 (3.8)	13 (2.1)	702 (3.3)	244 (5.7)	4,051 (3.2)
Scalp or neck	1,562 (6.3)	3,523 (8.9)	1,925 (7.8)	975 (10.4)	71 (11.4)	1,903 (9.1)	463 (10.7)	10,422 (8.4)
Lip	182 (0.7)	436 (1.1)	125 (0.5)	66 (0.7)	<3	208 (1.0)	>44	1,070 (0.9)
Eye	288 (1.2)	536 (1.3)	171 (0.7)	93 (1.0)	>3	205 (1.0)	<53	1,349 (1.1)
**Body (Trunk, lower extremity and upper extremity combined**)	13,565 (54.6)	15,279 (38.4)	14,843 (59.8)	4,354 (46.5)	340 (54.5)	8,151 (38.8)	1,575 (36.5)	58,107 (46.6)
Trunk	9,411 (37.9)	9,259 (23.3)	10,036 (40.4)	2,447 (26.1)	187 (30.0)	4,495 (21.4)	891 (20.7)	36,726 (29.4)
Lower extremity	1,867 (7.5)	2,851 (7.2)	2,213 (8.9)	868 (9.3)	70 (11.2)	1,803 (8.6)	302 (7.0)	9,974 (8.0)
Upper extremity	2,287 (9.2)	3,169 (8.0)	2,594 (10.5)	1,039 (11.1)	83 (13.3)	1,853 (8.8)	382 (8.9)	11,407 (9.1)
**Number of prior procedures done in dermatological specialties (IQR**)	0 (0–0)	0 (0–0)	0 (0–0)	0 (0–0)	17 (13–25)	0 (0–0)	0 (0–0)	0 (0–0)
**Number of tumours referred to other specialities (plastic, oncological or other departments), *n*(%**)	4,426 (17.8%)	7,782 (19.6%)	3,854 (15.5%)	1,747 (18.7%)	144 (23.1%)	4,397 (20.9%)	1,014 (23.5%)	23,364 (18.7%)

**Table III.** Comorbidities and medication prior inclusion date

	Cluster 1	Cluster 2	Cluster 3	Cluster 4	Cluster 5	Cluster 6	Cluster 7	Overall
**Medication, *n*(%**)								
Immunosuppressive drugs	804 (3.4)	1,249 (3.1)	582 (5.5)	542 (6.7)	67 (15.3)	2,125 (10.6)	544 (13.4)	5,913 (5.6)
Prednisolone	3,044 (12.9)	6,259 (15.7)	2,052 (19.5)	1,774 (22.1)	136 (31.1)	6,855 (34.1)	1,461 (36.1)	21,581 (20.3)
Ciclosporin	43 (0.2)	54 (0.1)	35 (0.3)	26 (0.3)	15 (3.4)	85 (0.4)	38 (0.9)	296 (0.3)
Azathioprine	274 (1.2)	234 (0.6)	125 (1.2)	153 (1.9)	24 (5.5)	446 (2.2)	90 (2.2)	1,346 (1.3)
Methotrexate	445 (1.9)	812 (2.0)	350 (3.3)	314 (3.9)	44 (10.1)	1,379 (6.9)	242 (6.0)	3,586 (3.4)
**Medication related to actinic keratosis or skin cancer, *n*(%**)								
Imiquimod	63 (0.3)	0 (0.0)	352 (3.3)	6,647 (82.7)	210 (48.1)	3 (0.0)	196 (4.8)	7,471 (7.0)
Fluorouracil cream	5 (0.0)	0 (0.0)	8 (0.1)	525 (6.5)	13 (3.0)	0 (0.0)	9 (0.2)	560 (0.5)
PDT	10 (0.0)	0 (0.0)	26 (0.2)	1,461 (18.2)	228 (52.2)	0 (0.0)	35 (0.9)	1,760 (1.7)
Summation of Imiquimod, fluorouracil cream and PDT, median (IQR)	0 (0–0)	0 (0–0)	0 (0–0)	1 (1–1)	1 (1–2)	0 (0–0)	0 (0–0)	0 (0–0)
**Skin disease, *n*(%**)								
Total number of skin cancer prior, median (IQR)	0 (0–0)	0 (0–0)	0 (0–0)	0 (0–2)	6 (3–13)	0 (0–0)	0 (0–0)	0 (0–0)
Melanoma	549 (2.3)	985 (2.5)	396 (3.8)	423 (5.3)	65 (14.9)	867 (4.3)	269 (6.6)	3,554 (3.3)
Nonmelanoma skin cancer	1,485 (6.3)	4,513 (11.4)	2,283 (21.7)	2,989 (37.2)	379 (86.7)	3,487 (17.3)	714 (17.6)	15,850 (14.9)
Actinic keratosis	182 (0.8)	362 (0.9)	170 (1.6)	1,102 (13.7)	256 (58.6)	374 (1.9)	121 (3.0)	2,567 (2.4)
**Comorbidities**								
Modified Charlson Comorbidity Index, mean (SD)	0.2 (0.5)	0.3 (0.7)	0.2 (0.7)	0.5 (1.0)	1.0 (2.1)	1.1 (1.1)	6.4 (2.1)	0.7 (1.5)
Diabetes, *n*(%)	134 (0.6)	594 (1.5)	84 (0.8)	154 (1.9)	12 (2.7)	806 (4.0)	992 (24.5)	2,776 (2.6)
Myocardial infarction, *n*(%)	172 (0.7)	1,073 (2.7)	135 (1.3)	252 (3.1)	6 (1.4)	1,144 (5.7)	742 (18.3)	3,524 (3.3)
Congestive heart failure, *n*(%)	84 (0.4)	638 (1.6)	114 (1.1)	189 (2.4)	16 (3.7)	1,126 (5.6)	893 (22.1)	3,060 (2.9)
Peripheral vascular disease, *n*(%)	174 (0.7)	908 (2.3)	125 (1.2)	236 (2.9)	15 (3.4)	1,357 (6.7)	860 (21.2)	3,675 (3.5)
Cancer, *n*(%)	775 (3.3)	2,483 (6.2)	594 (5.6)	735 (9.1)	46 (10.5)	4,385 (21.8)	1,994 (49.2)	11,012 (10.3)
Metastases, *n*(%)	<3	0 (0.0)	0 (0.0)	<8	22 (5.0)	0 (0.0)	925 (22.8)	951 (0.9)
Kidney disease, *n*(%)	4 (0.0)	0 (0.0)	0 (0.0)	19 (0.2)	14 (3.2)	0 (0.0)	1,494 (36.9)	1,531 (1.4)
Leukaemia, *n*(%)	62 (0.3)	133 (0.3)	42 (0.4)	75 (0.9)	8 (1.8)	239 (1.2)	280 (6.9)	839 (0.8)
Lymphoma, *n*(%)	74 (0.3)	143 (0.4)	48 (0.5)	53 (0.7)	4 (0.9)	255 (1.3)	318 (7.9)	895 (0.8)
**Smoking ever, *n*(%**)	2,175 (9.2)	4,320 (10.9)	1,335 (12.7)	1,070 (13.3)	78 (17.8)	4,964 (24.7)	1,207 (29.8)	15,149 (14.2)
**Alcohol abuse, *n*(%**)	640 (2.7)	1,494 (3.8)	396 (3.8)	271 (3.4)	17 (3.9)	1,131 (5.6)	310 (7.7)	4,259 (4.0)

### Cluster characteristics

**Fig. 1.**
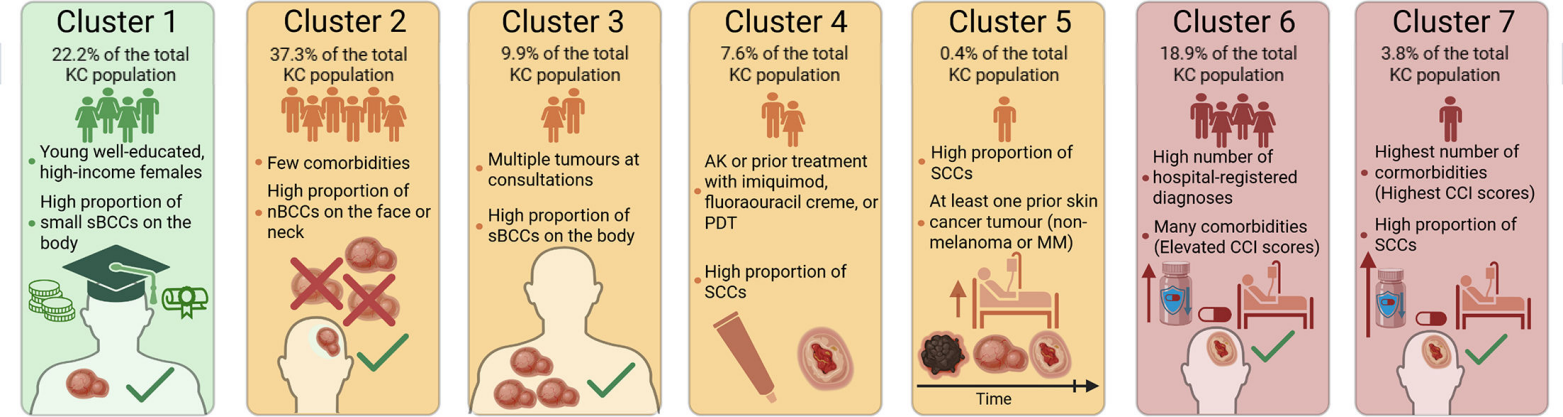
Overview of the 7 machine learning–derived clusters, coloured by clinical interpretation: medically and dermatologically noncomplex patients (green), dermatologically complex patients (orange) and medically and dermatologically complex patients (red). Created in BioRender. Sieborg, J. (2026) https://BioRender.com/5jld2qf.

A total of 7 patient clusters were identified (Fig. 1) by a combination of the elbow method, silhouette score and cluster stability score evaluation and were primarily distinguished by comorbidity, medication use and KC disease burden. The specific evaluation scores and figures, as well as the selection of the 7-cluster solution, are presented in greater detail in the Materials S1.

#### Medically and dermatologically noncomplex patients (cluster 1)

**Fig. 2.**
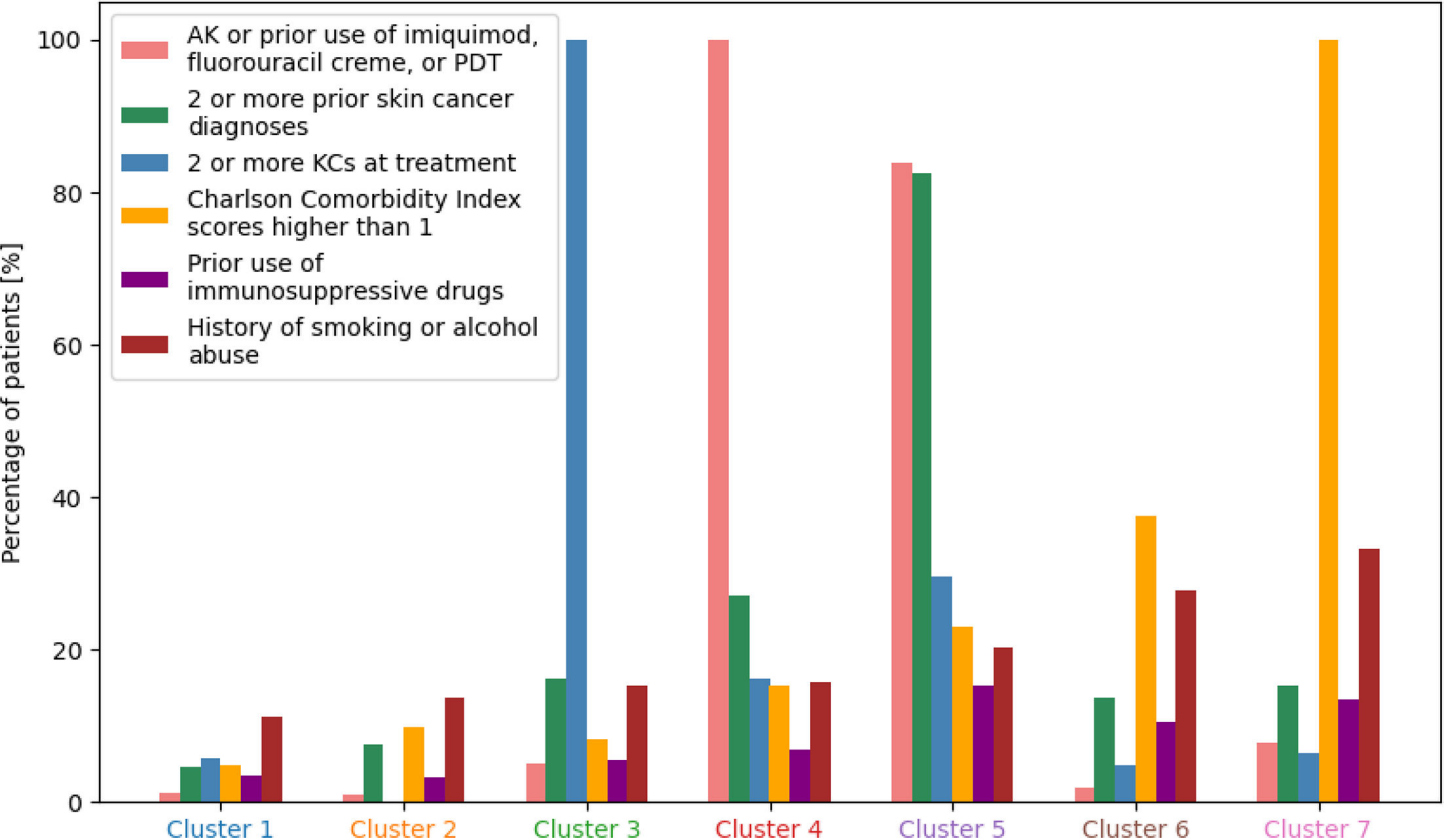
Histogram over percentage of different comorbidities in each cluster.

Cluster 1 (22.2% of KC population) was predominantly female (61.2%), a median 17.2 years younger than the total population, belonged to a high-income bracket, and were highly educated (55.6%). This first cluster’s KC tumours were almost exclusively BCCs (95.1% of their KCs), with a greater relative proportion of superficial BCC (sBCC; 21.2%) than most clusters. Their KCs were typically smaller than other clusters (33.1% less than 5 mm) and often located on the body (54.6%). The number of tumours (17.8%) referred to other medical specialties was similar to the overall KC population’s referral proportion. Reflecting the minimal morbidity of cluster 1, low usage of immunosuppressive drugs (3.4%), history of melanoma (2.3%), smoking (9.2%), alcohol abuse (2.7%) and modified mean CCI scores (0.2) was observed (Fig. 2) .

#### Patients with a greater dermatological disease burden (clusters 2–5)

Patients in clusters 2–5 were primarily patients with a greater dermatological disease burden and accounted for 55.2% of the KC population. These clusters included patients who had head and neck BCCs, multiple KC tumours at index visit, previous AK diagnoses or AK-related treatments and many with previous skin cancers.

Specifically, patients in cluster 2 (37.3% of KC population) most often presented with a single, slightly larger KC at the index visit, typically BCCs (86.7%). Additionally, their tumours were often located on head and neck (61.6%). The proportion of tumours referred to other medical specialties was 19.6% similar to the overall KC population. Patients in this cluster were more often male (58.6%), vocationally educated (48.5%) and demonstrated low levels of morbidity, including low usage of immunosuppressive drugs (3.1%), history of melanoma (2.5%), smoking history (10.9%) and low modified mean CCI score (0.3).

In contrast to cluster 2, where patients predominantly presented with a single KC, cluster 3 (9.9% of KC population) presented with multiple KCs at the index visit (median 2 tumours; IQR: 2–3). These KCs were most often on the body (59.8% of their KCs). The cluster furthermore had the highest relative proportion of sBCCs (24.9%) and few tumours were referred to other medical specialties (15.5%).

**Fig. 3.**
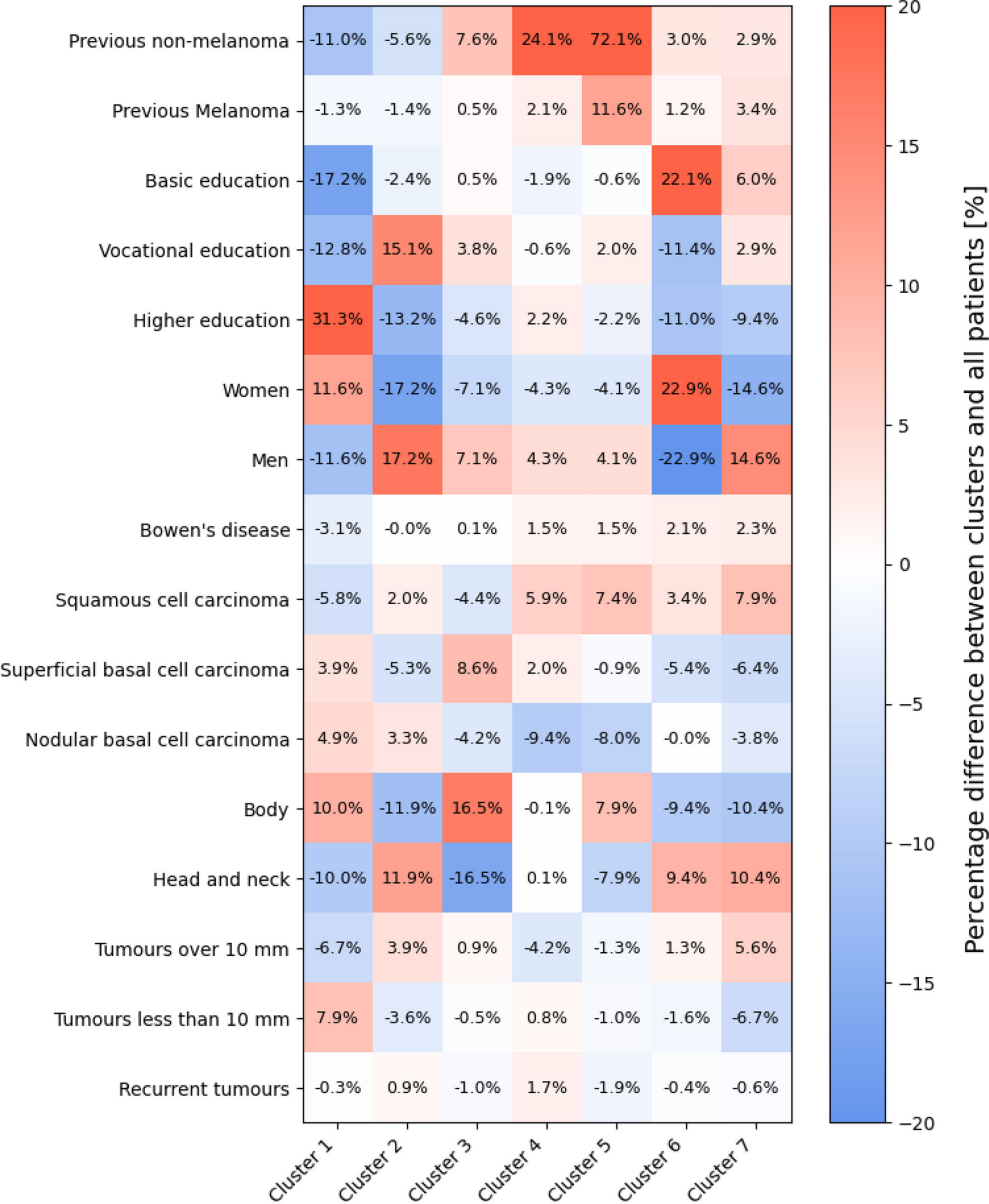
Heatmap over percentage point difference between each cluster compared with the rest of the KC population.

While cluster 3 was dominated by a high proportion of BCCs, cluster 4 (7.6%) conversely had many SCCs; 5.9 higher percentage points compared with the rest of the KC population (Fig. 3). This cluster was further characterized by high use of PDT, imiquimod or 5-fluorouracil therapy, as well as a relatively high frequency of hospital-registered AK diagnoses (13.7%), potentially indicating the highest degree of actinic skin damage and field cancerization among the identified clusters.

Cluster 5 was a particularly small segment (0.4%) primarily distinguished by many SCCs (14.7%) and high frequency of referral to other medical specialties (23.1%). Further, a large proportion of patients in this cluster had melanoma (14.9%) or nonmelanoma skin cancer (86.7%) registered in their medical history prior to index date. These patients also had a more frequent history of skin cancer, AK, and use of immunosuppressive drugs compared to the overall KC population.

#### Medically complex patients with elevated dermatological disease burden (clusters 6 & 7)

The remaining quarter of the total KC population (22.7%) belonged to clusters 6 and 7, which was comprised of medically complex, older and more vulnerable patients. These patients had higher modified CCI scores, high medication use, many hospital-registered diagnoses, and relatively many with a history of alcohol and smoking abuse compared to cluster 1–5.

The larger of the 2, cluster 6 (18.9%), had a high proportion of women with basic educational attainment, many hospital-registered diagnoses, and more medical comorbidities compared to clusters 1–4 (one third had >1 modified CCI scores). A slightly higher proportion of these patients” KCs were SCCs (10.1%) often located on head and neck (61.2%).

In the smaller cluster 7 (3.8%), a high prevalence of SCCs was observed (15.0%) also primarily located on head and neck (63.5%). Consistent with this, patients in this cluster had the highest proportion of KCs referred to other medical specialties (23.5%). Additionally, many patients in this cluster have had melanoma (6.6%) prior index date. Although these individuals had comparable numbers of hospital-registered diagnoses to cluster 6, patients had substantially more comorbidities, reflected by consistently elevated modified CCI scores above 1 and a mean of 6.4, and were predominantly older males.

## DISCUSSION

This nationwide registry-based study of over 100,000 unique patients in office-based dermatological practices distinguished 7 KC patient subgroups in the Danish KC population using a data-driven ML approach. The identified phenogroups, with their differing health statuses, KC-related risk profiles, current disease burden, and SES, appear clinically meaningful as these factors may influence past and prospective sun exposure, health literacy, care utilization, and ability to attend follow-up (26–29). Together, these findings suggest that phenomapping can be applied to the KC population to identify unique patient profiles.

Interestingly, the study identified a large patient cluster that was mostly medically noncomplex with limited disease and few comorbidities (cluster 1). This cluster commonly had small BCCs located on the body and consisted of young, highly educated, high-income, mostly healthy noncomplex females. It is commonly observed that younger BCC patients are more often female (30, 31), and that higher SES is associated with an increased incidence (13, 14); these established patterns could thus be reproduced using the ML approach. Although many of these medically noncomplex patients have low risk BCCs, nearly one fifth (17.9%) of their tumours are still referred to other medical specialties beside office-based dermatological practices. This suggests that other factors are influencing management decisions. In general, the cluster represents a relatively healthy patient subgroup that might generally be expected adequately and cost-effectively treated outside the hospital setting, as many of their KCs are low-risk BCCs.

Another cluster with few comorbidities consisted primarily of vocationally educated males (cluster 2). Patients in this cluster often presented a single head and neck nBCC; an anatomical location considered high risk. Additionally, patients in this cluster did have slightly larger tumours and a higher proportion of SCCs compared with other KC patients. However, these patients were mostly medically noncomplex with few comorbidities and limited history of AK, skin cancer or related treatments. Given their low medical complexity, this cluster may, like cluster 1, be adequately managed outside the hospital. On the other hand, it remains possible that other factors not captured by the model might inform management decisions.

Evidence suggests that many individuals go on to develop additional KCs following their first KC tumour (32, 33). Cluster 3 reflected this clinical phenomenon, consisting of dermatologically complex patients with multiple KCs at consultation. This cluster may potentially also include patients that postpone healthcare check-ups, thereby allowing multiple KCs to develop. The second explanation could explain the limited record of skin-related comorbidities for this cluster. Most KCs in this subgroup were BCCs located on the body, and while not difficult to treat individually, the substantial number adds complexity to their dermatological management.

Approximately 7.8% of patients (cluster 4–5) had multiple skin-related comorbidities. As such, almost all patients had received dermatological care, consisting of either previous AK-related treatment or dermatological procedures (SKS: KQ* or KYQ*). Consistent with a high prevalence of AK and history of AK-related treatments, a larger proportion of their tumours were SCCs. Compared to clusters 1 and 2, clusters 4 and 5 might be more often appropriately allocated to specialized hospital care, in light of their greater dermatological complexity and KC risk level (sun damage and prevalence of the high-risk KC subtype SCC).

A considerable percentage of patients had close contact with the healthcare system with multiple hospital-registered diagnoses, multiple comorbidities, high prevalence of SCCs on head and neck, along with considerable prior use of immunosuppressive drugs (clusters 6 and 7). Treatment of these medically complex patients is often less straightforward, as some treatment modalities can be limited by comorbidity, competing medical considerations, and individual physical/mental/socioeconomic resources. Further, increased monitoring frequency may be required to address the increased risk for developing multiple KCs due to immunosuppressive drugs (5). In particular, the individuals in cluster 7 with high modified CCI scores also had numerous previous skin cancers and a substantial number of SCCs, frequently located on head and neck and of larger tumour size. In tailoring clinical pathways for such patient subsets, specialized, hospital-based dermatological care and follow-up may more often be indicated to address their expanded medical and dermatological needs.

Real-world data are difficult to cluster, and clusters often overlap in patient characteristics (22, 34). Hence, selecting the optimal number yielding distinct reproducible and clinically meaningful clusters can be challenging. Alternative solutions (2, 3, and 6 clusters) are presented in the Supplementary Materials 1–3. The 6-cluster solution compared to the 7-cluster solution had marginally better performance based on the elbow method, silhouette score (50,000 sample) and cluster stability, but the 7-cluster solution was ultimately selected due to its ability to capture a clinically meaningful distinction between individuals with highly elevated versus moderately elevated CCI scores. Importantly, several clusters (clusters 3–5, and to some extent clusters 1 and 2) were consistent across both solutions, supporting the robustness and stability of these subgroup structures.

Study limitations include incomplete and missing data from the registries which could potentially lead to selection bias if data is not missing at random (24, 35). Suboptimal completeness would presumably not change the identified clusters; however, it could affect the distribution of individuals in the clusters if missing patients share specific characteristics. Importantly, this study did not assess differences in clinical outcomes such as recurrence or complications for the identified clusters. It therefore remains to be confirmed whether the clusters are clinically meaningful, and clinical interpretations can be influenced by observer bias. Furthermore, determining the optimal number of clusters is inherently challenging, particularly in real-world data where the boundaries between patient groups are often diffuse. Nonetheless, the study also has several strengths, including the size and granularity of the Danish Skin Cancer Registry, which contains data on over 100,000 unique KC patients spanning almost a decade. The Danish registries allowed evaluation of not only patient-specific information but also of tumour characteristics, enabling each patient cluster to be assessed based on medical history, comorbidities, patient relevant features, and current KC disease burden. Additionally, even though the KC subtype, tumour size, and anatomical location were not included in the ML clustering, differences in tumour characteristics between the clusters were still apparent, suggesting that distinct risk profiles may be associated with varying KC burdens.

In conclusion, the KC population consisted of 7 clusters from an ML-driven approach, each with distinct phenotypic characteristics. Crucially, the multidimensional analysis revealed dermatologically and medically complex patient clusters that potentially may benefit from specialized care and monitoring pathways. Integrating these clusters into a more personalized, patient-centric management strategy could not only improve individual outcomes but also improve the efficiency of KC care pathways, which is important for handling the growing KC burden (3). These findings represent an important step toward more personalized patient-centric management and could help provide a foundation for tailored clinical pathways based on different resource needs.

## Data Availability

The data underlying this study cannot be shared publicly as Danish law does not allow transfer of these data.
