# Supplementary material for: Machine-learning-based Phenomapping of Patients with Keratinocyte Carcinoma: Data-driven Subgrouping by Disease Burden, Comorbidities and Socioeconomic Status
Source: Acta Derm Venereol. 2026 Aug 13;106:0843. doi: 10.2340/actadv.v106.adv-2026-0843 (PMC13470789; doi:10.2340/actadv.v106.adv-2026-0843)
Supplement: Supplementary file 1 [file ActaDv-106-0843-s0001.pdf]

Appendix S1

SUPPLEMENTARY MATERIALS

Immunosuppressive drugs

Immunosuppressive drugs included the Danish health care classification system codes (Sundhedsvæsenets Klassifikationssystem, SKS) ML04* and BOHJ* (excluding antibody treatments) and Anatomical Therapeutic Chemical Classification System (ATC) codes L04*. Prednisolone included the SKS codes (MA07EA01, MH02AB06, MC05AA04, MD07AA03, MD07XA02, MR01AD02, MS01CB02, MS01BA04, MS02BA03, MS01CB02, MS03BA02, MR01AD52, MA01AC54, MD07BA01, MD07CA03, MS01CA02, MV03AB05, BWHA159, BWHA171, BWHA134, BWHA137, BWHA168) and ATC codes (H02AB06 and A07AE01). Methotrexate included the SKS codes (BWHA115, BWHA122, BWHA130, BWHA117, BWHA161, BWHA185, BWHA219, BKHE8A) and ATC codes (L01BA01 and L04AX03). Azathioprine included SKS codes (BWHB83) and ATC codes (L04AX01). Ciclosporin included SKS codes (BOHJ20) and ATC codes (L04AD01 and S01XA18). Imiquimod included SKS (MD06BB10 and BNHG01A) and ATC (D06BB10). Photodynamic therapy (PDT) included SKS (BWHD and BNGC1) and ATC (L01XD03 and L01XD04). Fluorouracil cream included the ATC (L01BC02).

Determining the number of clusters.

To determine the number of clusters, three different evaluation metrics were used: the elbow method, the silhouette score and cluster stability. The elbow method was estimated on the whole dataset while the silhouette score was estimated from a 50,000 random sample of the population due to high computation time of calculating the distance matrix (Figure S1 and S2). The cluster stability was assessed via bootstrapping (20 iterations), with the mean Adjusted Rand Index (ARI) calculated for each number of clusters (Figure S3). Based on these three-evaluation metrics, excluding choices with 2 and 3 clusters, the optimal number of clusters was determined to be 6 or 7. Seven clusters were selected over six clusters due to the finer distinction between those with high comorbidity score and moderate comorbidity score. The 6-cluster choice and corresponding figures (S4 and S5) and tables (Table S1–S3) are presented below. This solution reproduces similar cluster to the 7-cluster choice, especially cluster 3–5, which are almost identical between both cluster choices. Furthermore, cluster 1 in the 6-cluster choice was young, high-income individuals with high proportion of BCCs, similarly to cluster 1 in the 7-cluster solution, however, did differentiate by having slightly more males than females in contrast to cluster 1 in the 7-cluster choice. The 6-cluster choice only has one highly comorbid cluster and a much larger cluster 2.

The 2- and 3-cluster choice

The 2 and 3 cluster choices were not chosen as the broad split did not represent the heterogenous keratinocyte carcinoma patient group well and the clinical interpretation between the few clusters were less refined and clinically meaningful than the 6 or 7 cluster choice. Briefly, the 2-cluster choice mainly differentiated on younger and high-income patients versus older and lower-income individuals. The 3-cluster choice mainly differentiated again on a younger, high-income patient cluster versus two older patient clusters which differentiated from each other on number of prior skin cancers before index.

Figure S1: Showing the loss with respect to the number of clusters.

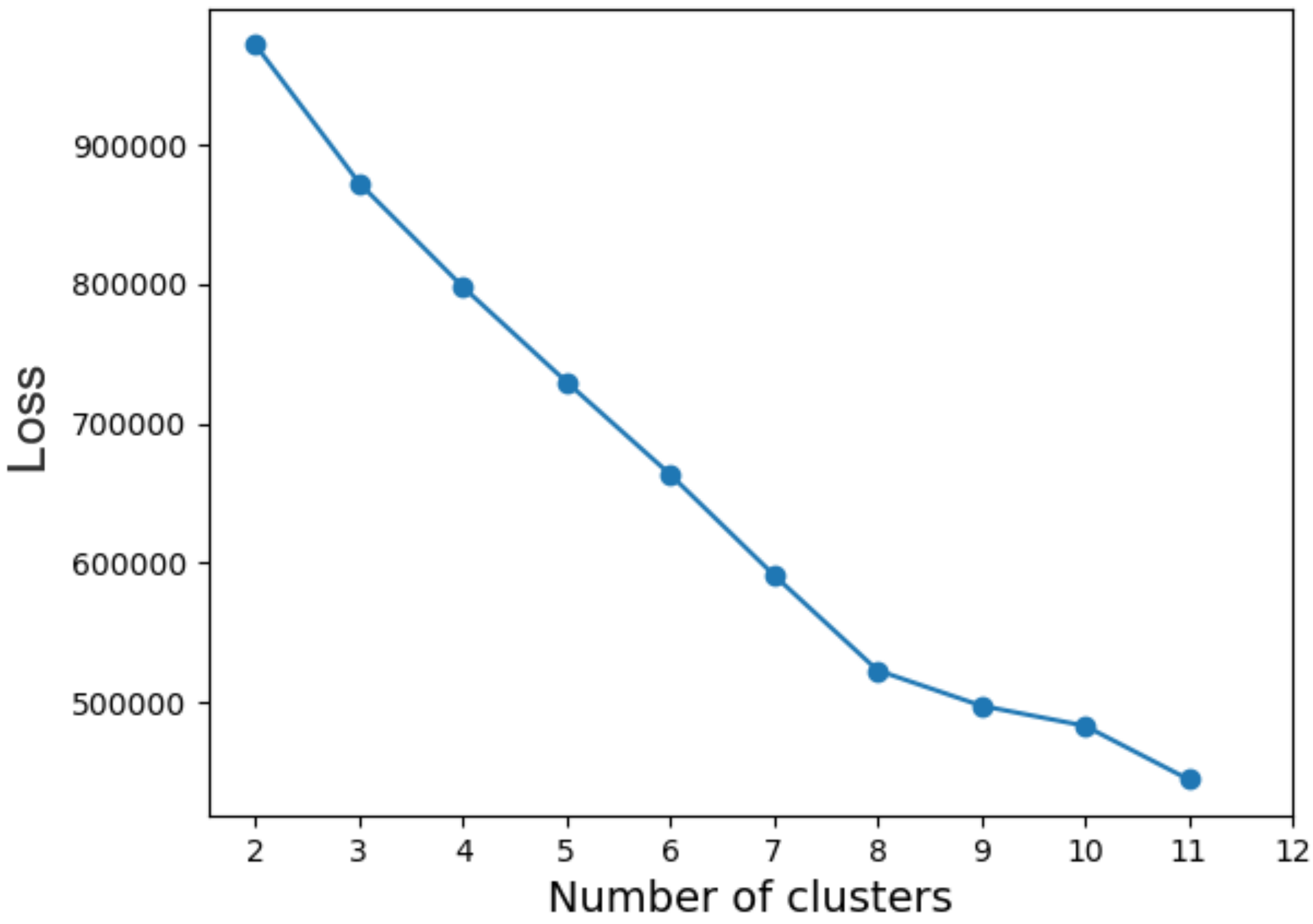

Figure S2: Showing the silhouette score with respect to the number of clusters estimated from a 50,000 random sample of the population.

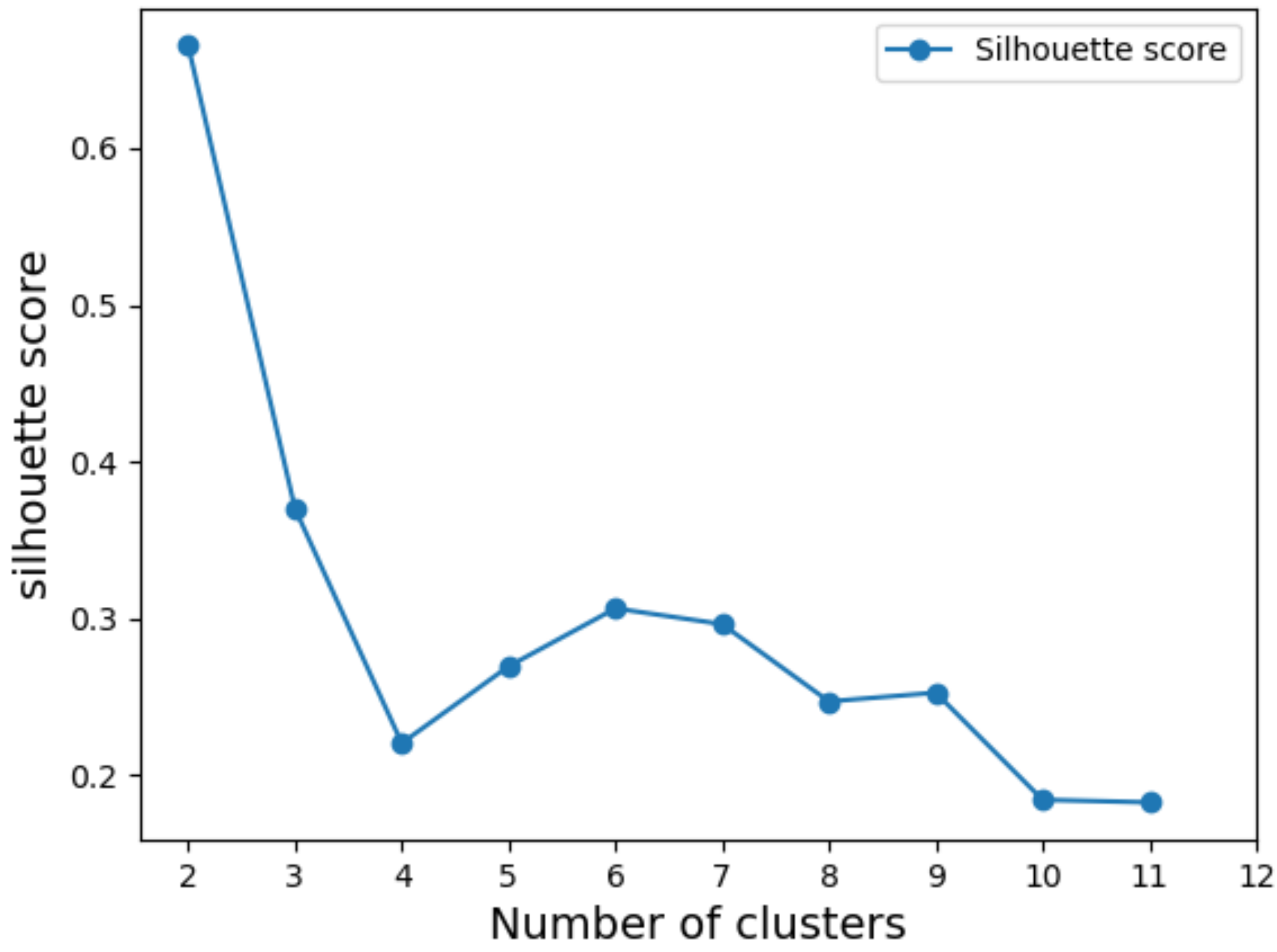

Figure S3: Showing the cluster stability with respect to the number of clusters using adjusted rand index as cluster stability score.

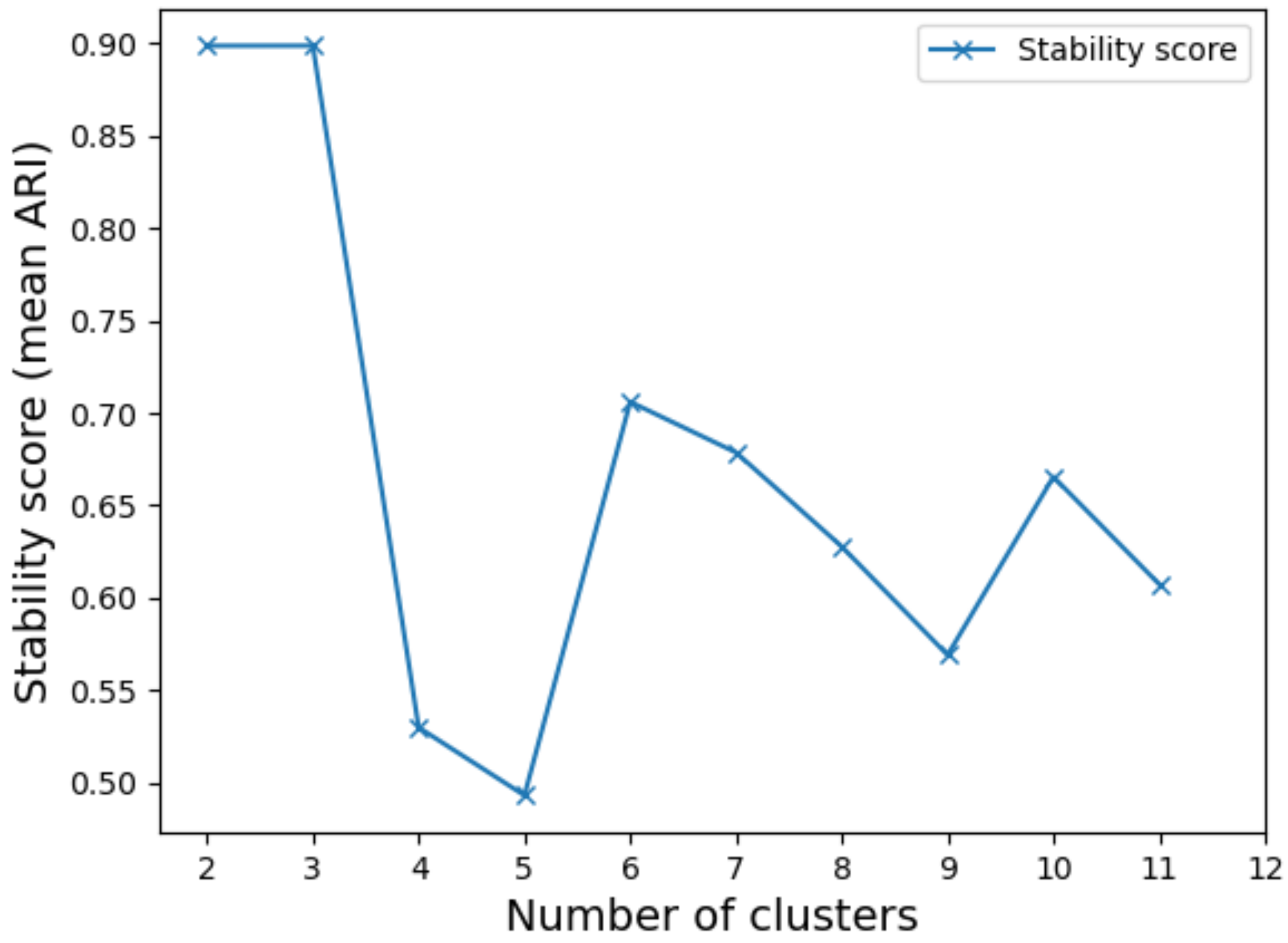

Stability score
Stability score (mean ARI)
0.90
0.85
0.80
0.75
0.70
0.65
0.60
0.55
0.50
2
3
4
5
6
7
8
9
10
11
12
Number of clusters

Figure S4: Histogram over percentage of different comorbidities in each cluster for the 6-cluster solution.

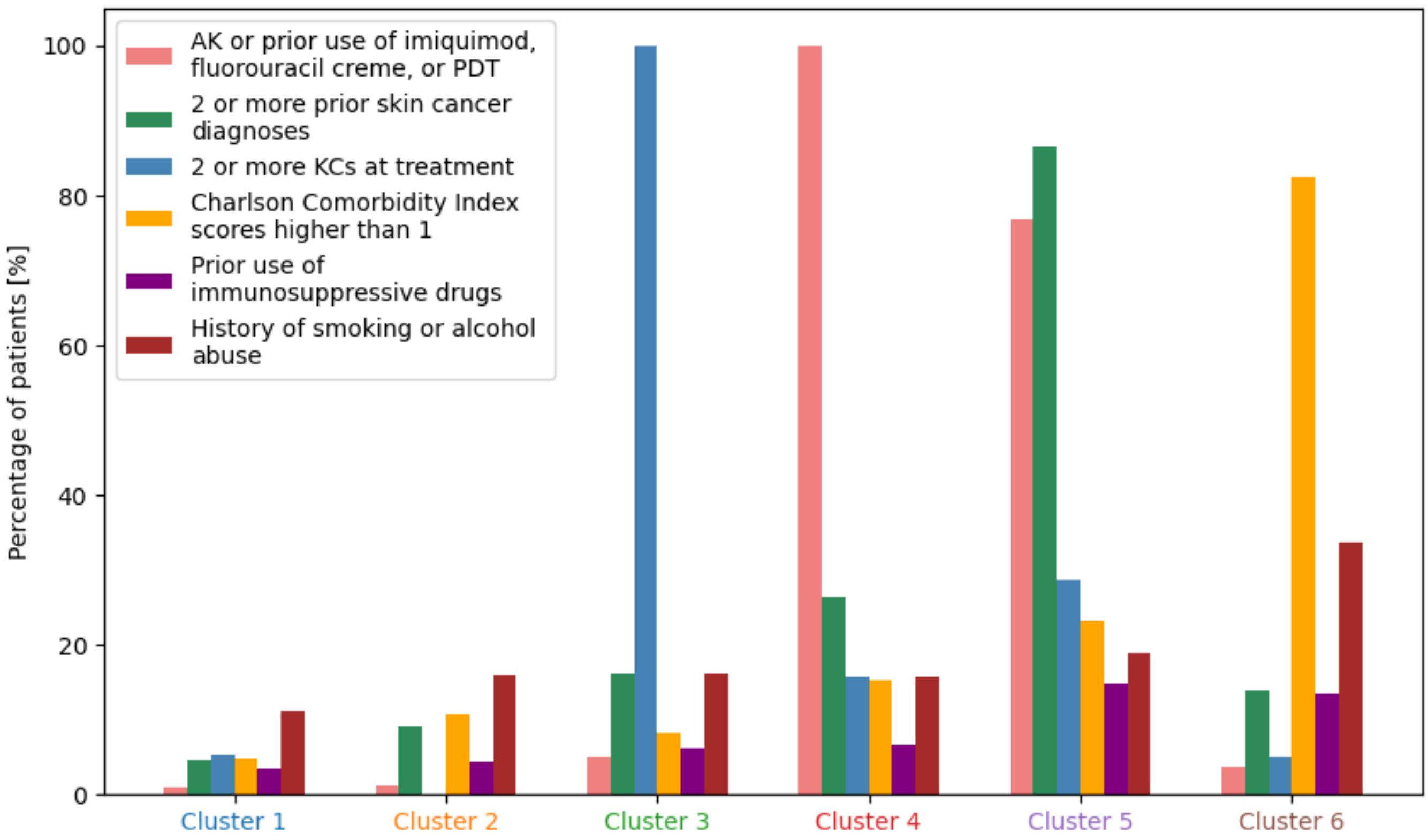

Figure S5: Heatmap over percentage point difference between each cluster compared with the rest of the KC population for the 6-cluster solution.

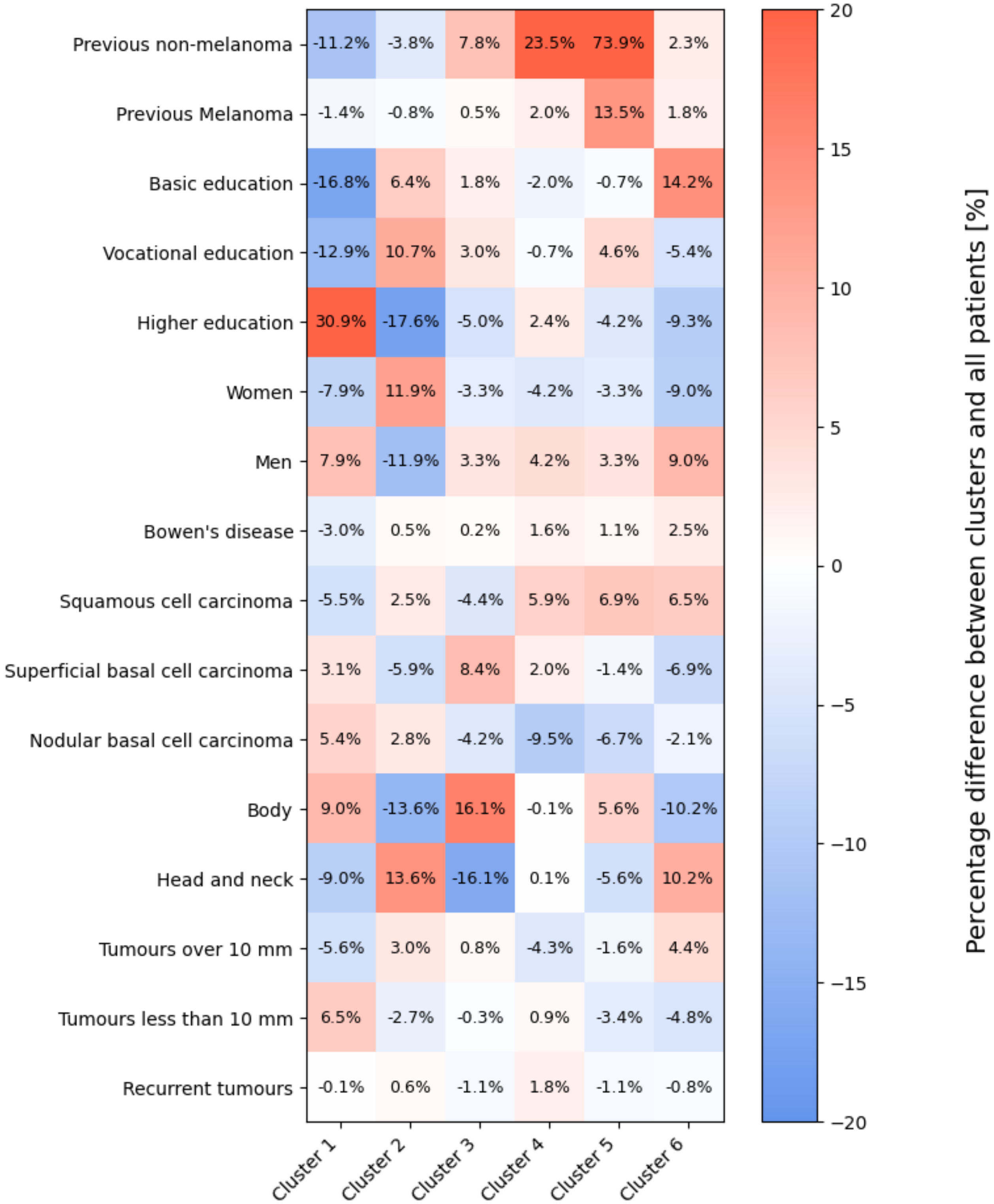

Table S1: Patient characteristics for the 6-cluster choice

	Cluster 1	Cluster 2	Cluster 3	Cluster 4	Cluster 5	Cluster 6	Overall
**Total number of patients**	25590 (24.0%)	48994 (46.0%)	11082 (10.4%)	7993 (7.5%)	567 (0.5%)	12264 (11.5%)	106490 (100%)
**Sex, n(%)**							
Male	13779 (53.8)	20279 (41.4)	5627 (50.8)	4138 (51.8)	290 (51.1)	6842 (55.8)	50955 (47.8)
Female	11811 (46.2)	28715 (58.6)	5455 (49.2)	3855 (48.2)	277 (48.9)	5422 (44.2)	55535 (52.2)
**Age at treatment, median**	56.4 (49.1–63.6)	75.1 (69.9–80.9)	74.5 (68.0–80.8)	75.3 (68.4–81.3)	76.6 (70.8–83.0)	77.9 (72.1–83.7)	72.5 (63.5–79.4)
**Disposable Income [DKK] at index date, median**	788632 (574183–	319826 (237336–	348485 (252306–	387331 (274282–	338749 (254429–	324127 (238801–	387567 (266729–
**Number of hospital visits**	11 (6–20)	17 (10–27)	18 (9–30)	21 (12–34)	39 (23–58.5)	48 (34–65)	18 (9–31)
**Highest achieved education, n(%)**							
Higher education	14019 (54.8)	10679 (21.8)	2968 (26.8)	2682 (33.6)	154 (27.2)	2831 (23.1)	33333 (31.3)
Basic education	3916 (15.3)	15449 (31.5)	3288 (29.7)	2096 (26.2)	155 (27.3)	4982 (40.6)	29886 (28.1)
Vocational education	7469 (29.2)	21941 (44.8)	4618 (41.7)	3064 (38.3)	247 (43.6)	4200 (34.2)	41539 (39.0)
Unknown education	186 (0.7)	925 (1.9)	208 (1.9)	151 (1.9)	11 (1.9)	251 (2.0)	1732 (1.6)

Table S2: Tumour characteristics for the 6-cluster choice

Table S3: Comorbidities and medication prior inclusion date for 6-cluster choice

	Cluster 1	Cluster 2	Cluster 3	Cluster 4	Cluster 5	Cluster 6	Overall
**Medication, n(%)**							
Immunosuppressive drugs	880 (3.4)	2108 (4.3)	673 (6.1)	530 (6.6)	84 (14.8)	1638 (13.4)	5913 (5.6)
Prednisolone	3278	9379 (19.1)	2312 (20.9)	1748 (21.9)	168 (29.6)	4696 (38.3)	21581
Ciclosporin	47 (0.2)	92 (0.2)	39 (0.4)	25 (0.3)	16 (2.8)	77 (0.6)	296 (0.3)
Azathioprine	310 (1.2)	420 (0.9)	144 (1.3)	148 (1.9)	31 (5.5)	293 (2.4)	1346 (1.3)
Methotrexate	456 (1.8)	1444 (2.9)	407 (3.7)	311 (3.9)	54 (9.5)	914 (7.5)	3586 (3.4)
**Medication related to actinic keratosis or skin cancer, n(%)**							
Imiquimod	52 (0.2)	0 (0.0)	365 (3.3)	6618 (82.8)	251 (44.3)	185 (1.5)	7471 (7.0)
Fluorouracil cream	5 (0.0)	0 (0.0)	8 (0.1)	523 (6.5)	16 (2.8)	8 (0.1)	560 (0.5)
PDT	5 (0.0)	0 (0.0)	27 (0.2)	1433 (17.9)	265 (46.7)	30 (0.2)	1760 (1.7)
Summation of Imiquimod, fluorouracil	0 (0–0)	0 (0–0)	0 (0–0)	1 (1–1)	1 (0–1)	0 (0–0)	0 (0–0)
**Skin disease, n(%)**							
Total number of skin cancer prior,	0 (0–0)	0 (0–0)	0 (0–0)	0 (0–2)	8 (4–18)	0 (0–0)	0 (0–0)
Melanoma	587 (2.3)	1435 (2.9)	423 (3.8)	414 (5.2)	95 (16.8)	600 (4.9)	3554 (3.3)
Non-melanoma skin cancer	1637 (6.4)	6290 (12.8)	2424 (21.9)	2926 (36.6)	501 (88.4)	2072 (16.9)	15850
Actinic keratosis	197 (0.8)	541 (1.1)	185 (1.7)	1077 (13.5)	292 (51.5)	275 (2.2)	2567 (2.4)
**Comorbidities**							
Modified Charlson Comorbidity Index,	0.2 (0.5)	0.4 (0.7)	0.2 (0.7)	0.5 (1.0)	1.1 (2.2)	3.4 (2.5)	0.7 (1.5)
Diabetes, n(%)	167 (0.7)	701 (1.4)	86 (0.8)	153 (1.9)	16 (2.8)	1653 (13.5)	2776 (2.6)
Myocardial infarction, n(%)	242 (0.9)	1239 (2.5)	135 (1.2)	248 (3.1)	14 (2.5)	1646 (13.4)	3524 (3.3)
Congestive heart failure, n(%)	121 (0.5)	813 (1.7)	125 (1.1)	184 (2.3)	23 (4.1)	1794 (14.6)	3060 (2.9)
Peripheral vascular disease, n(%)	190 (0.7)	1206 (2.5)	128 (1.2)	234 (2.9)	19 (3.4)	1898 (15.5)	3675 (3.5)
Cancer, n(%)	839 (3.3)	3401 (6.9)	629 (5.7)	720 (9.0)	59 (10.4)	5364 (43.7)	11012
Metastases, n(%)	<3	0 (0.0)	<3	>11	32 (5.6)	897 (7.3)	951 (0.9)
Kidney disease, n(%)	5 (0.0)	0 (0.0)	<3	>33	16 (2.8)	1472 (12.0)	1531 (1.4)
Leukaemia, n(%)	69 (0.3)	150 (0.3)	45 (0.4)	75 (0.9)	12 (2.1)	488 (4.0)	839 (0.8)
Lymphoma, n(%)	86 (0.3)	156 (0.3)	44 (0.4)	50 (0.6)	6 (1.1)	553 (4.5)	895 (0.8)
**Smoking ever, n(%)**	2224 (8.7)	6607 (13.5)	1491 (13.5)	1056 (13.2)	94 (16.6)	3677 (30.0)	15149
**Alcohol abuse, n(%)**	831 (3.2)	1793 (3.7)	439 (4.0)	271 (3.4)	22 (3.9)	903 (7.4)	4259 (4.0)
