# Supplementary material for: Machine-learning-based Phenomapping of Patients with Keratinocyte Carcinoma: Data-driven Subgrouping by Disease Burden, Comorbidities and Socioeconomic Status
Source: Acta Derm Venereol. 2026 Aug 13;106:0843. doi: 10.2340/actadv.v106.adv-2026-0843 (PMC13470789; doi:10.2340/actadv.v106.adv-2026-0843)
Supplement: Supplementary file 2 [file ActaDv-106-0843-s0002.pdf]

Table SI. Patient characteristics for the 6-cluster choice

	Cluster 1	Cluster 2	Cluster 3	Cluster 4	Cluster 5	Cluster 6	Overall
**Total number of patients**	25590 (24.0%)	48994 (46.0%)	11082 (10.4%)	7993 (7.5%)	567 (0.5%)	12264 (11.5%)	106490 (100%)
**Sex, n(%)**							
Male	13779 (53.8)	20279 (41.4)	5627 (50.8)	4138 (51.8)	290 (51.1)	6842 (55.8)	50955 (47.8)
Female	11811 (46.2)	28715 (58.6)	5455 (49.2)	3855 (48.2)	277 (48.9)	5422 (44.2)	55535 (52.2)
**Age at treatment, median (IQR)**	56.4 (49.1–63.6)	75.1 (69.9–80.9)	74.5 (68.0–80.8)	75.3 (68.4–81.3)	76.6 (70.8–83.0)	77.9 (72.1–83.7)	72.5 (63.5–79.4)
**Disposable Income [DKK] at index date, median (IQR)**	788632 (574183–1063680)	319826 (237336–430953)	348485 (252306–511676)	387331 (274282–586005)	338749 (254429–521194)	324127 (238801–456013)	387567 (266729–605489)
**Number of hospital visits with diagnosis, median (IQR)**	11 (6–20)	17 (10–27)	18 (9–30)	21 (12–34)	39 (23–58.5)	48 (34–65)	18 (9–31)
**Highest achieved education, n(%)**							
Higher education	14019 (54.8)	10679 (21.8)	2968 (26.8)	2682 (33.6)	154 (27.2)	2831 (23.1)	33333 (31.3)
Basic education	3916 (15.3)	15449 (31.5)	3288 (29.7)	2096 (26.2)	155 (27.3)	4982 (40.6)	29886 (28.1)
Vocational education	7469 (29.2)	21941 (44.8)	4618 (41.7)	3064 (38.3)	247 (43.6)	4200 (34.2)	41539 (39.0)
Unknown education	186 (0.7)	925 (1.9)	208 (1.9)	151 (1.9)	11 (1.9)	251 (2.0)	1732 (1.6)

Table SII. Tumour characteristics for the 6-cluster choice

	Cluster 1	Cluster 2	Cluster 3	Cluster 4	Cluster 5	Cluster 6	Overall
Total number of tumours	26875 (21.5%)	48994 (39.3%)	25908 (20.8%)	9271 (7.4%)	795 (0.6%)	12883 (10.3%)	124726 (100%)
**Tumour diagnoses, n(%)**							
Nodular basal cell carcinoma	19950 (74.2)	35134 (71.7)	17270 (66.7)	5678 (61.2)	504 (63.4)	8777 (68.1)	87313 (70.0)
Superficial basal cell carcinoma	5518 (20.5)	7104 (14.5)	6409 (24.7)	1847 (19.9)	133 (16.7)	1536 (11.9)	22547 (18.1)
Bowen's disease	596 (2.2)	2407 (4.9)	1231 (4.8)	559 (6.0)	45 (5.7)	875 (6.8)	5713 (4.6)
Squamous cell carcinoma	811 (3.0)	4349 (8.9)	998 (3.9)	1187 (12.8)	113 (14.2)	1695 (13.2)	9153 (7.3)
Recurrent tumour	791 (2.9)	1689 (3.4)	574 (2.2)	439 (4.7)	16 (2.0)	304 (2.4)	3813 (3.1)
Number of tumours at treatment, median (IQR)	1 (1–1)	1 (1–1)	2 (2–3)	1 (1–2)	1 (1–2)	1 (1–1)	1 (1–2)
**Tumour size, n(%)**							
< 5 mm	8433 (31.4)	12338 (25.2)	7000 (27.0)	2692 (29.0)	214 (26.9)	2895 (22.5)	33572 (26.9)
5-10 mm	11126 (41.4)	19991 (40.8)	10457 (40.4)	3656 (39.4)	297 (37.4)	5269 (40.9)	50796 (40.7)
10-15 mm	3021 (11.2)	6734 (13.7)	3343 (12.9)	992 (10.7)	92 (11.6)	1935 (15.0)	16117 (12.9)
> 15 mm	1469 (5.5)	4503 (9.2)	2295 (8.9)	600 (6.5)	63 (7.9)	1292 (10.0)	10222 (8.2)
Unknown/irregular	2808 (10.4)	5382 (11.0)	2790 (10.8)	1320 (14.2)	127 (16.0)	1483 (11.5)	13910 (11.2)
**Anatomical locations, n(%)**							
**Head and neck (Face, ear, scalp or neck, lip, eye combined)**	12453 (46.3)	30219 (61.7)	10540 (40.7)	4962 (53.5)	380 (47.8)	8065 (62.6)	66619 (53.4)
Face	9554 (35.5)	22895 (46.7)	7681 (29.6)	3482 (37.6)	256 (32.2)	5859 (45.5)	49727 (39.9)
Ear	567 (2.1)	1924 (3.9)	512 (2.0)	358 (3.9)	20 (2.5)	670 (5.2)	4051 (3.2)

Scalp or neck	1839 (6.8)	4219 (8.6)	2030 (7.8)	963 (10.4)	94 (11.8)	1277 (9.9)	10422 (8.4)
Lip	186 (0.7)	552 (1.1)	130 (0.5)	<70	<3	134 (1.0)	1070 (0.9)
Eye	307 (1.1)	629 (1.3)	187 (0.7)	>89	>4	125 (1.0)	1349 (1.1)
**Body (Trunk, lower extremity and upper extremity combined)**	14422 (53.7)	18775 (38.3)	15368 (59.3)	4309 (46.5)	415 (52.2)	4818 (37.4)	58107 (46.6)
Trunk	10058 (37.4)	10964 (22.4)	10316 (39.8)	2418 (26.1)	231 (29.1)	2739 (21.3)	36726 (29.4)
Lower extremity	1836 (6.8)	3913 (8.0)	2350 (9.1)	851 (9.2)	86 (10.8)	938 (7.3)	9974 (8.0)
Upper extremity	2528 (9.4)	3898 (8.0)	2702 (10.4)	1040 (11.2)	98 (12.3)	1141 (8.9)	11407 (9.1)
**Number of prior procedures done in dermatological specialties (IQR)**	0 (0–0)	0 (0–0)	0 (0–0)	0 (0–0)	15 (8–22)	0 (0–0)	0 (0–0)
**Number of tumours referred to other specialities (plastic, oncological or other departments), n(%)**	4808 (17.9%)	9666 (19.7%)	4049 (15.6%)	1726 (18.6%)	190 (23.9%)	2925 (22.7%)	23364 (18.7%)

Table SIII. Comorbidities and medication prior inclusion date for 6-cluster choice

	Cluster 1	Cluster 2	Cluster 3	Cluster 4	Cluster 5	Cluster 6	Overall
**Medication, n(%)**							
Immunosuppressive drugs	880 (3.4)	2108 (4.3)	673 (6.1)	530 (6.6)	84 (14.8)	1638 (13.4)	5913 (5.6)
Prednisolone	3278 (12.8)	9379 (19.1)	2312 (20.9)	1748 (21.9)	168 (29.6)	4696 (38.3)	21581 (20.3)
Ciclosporin	47 (0.2)	92 (0.2)	39 (0.4)	25 (0.3)	16 (2.8)	77 (0.6)	296 (0.3)
Azathioprine	310 (1.2)	420 (0.9)	144 (1.3)	148 (1.9)	31 (5.5)	293 (2.4)	1346 (1.3)
Methotrexate	456 (1.8)	1444 (2.9)	407 (3.7)	311 (3.9)	54 (9.5)	914 (7.5)	3586 (3.4)
**Medication related to actinic keratosis or skin cancer, n(%)**							
Imiquimod	52 (0.2)	0 (0.0)	365 (3.3)	6618 (82.8)	251 (44.3)	185 (1.5)	7471 (7.0)
Fluorouracil cream	5 (0.0)	0 (0.0)	8 (0.1)	523 (6.5)	16 (2.8)	8 (0.1)	560 (0.5)
PDT	5 (0.0)	0 (0.0)	27 (0.2)	1433 (17.9)	265 (46.7)	30 (0.2)	1760 (1.7)
Summation of Imiquimod, fluorouracil cream and PDT, median (IQR)	0 (0–0)	0 (0–0)	0 (0–0)	1 (1–1)	1 (0–1)	0 (0–0)	0 (0–0)
**Skin disease, n(%)**							
Total number of skin cancer prior, median (IQR)	0 (0–0)	0 (0–0)	0 (0–0)	0 (0–2)	8 (4–18)	0 (0–0)	0 (0–0)
Melanoma	587 (2.3)	1435 (2.9)	423 (3.8)	414 (5.2)	95 (16.8)	600 (4.9)	3554 (3.3)
Non-melanoma skin cancer	1637 (6.4)	6290 (12.8)	2424 (21.9)	2926 (36.6)	501 (88.4)	2072 (16.9)	15850 (14.9)
Actinic keratosis	197 (0.8)	541 (1.1)	185 (1.7)	1077 (13.5)	292 (51.5)	275 (2.2)	2567 (2.4)
**Comorbidities**							
Modified Charlson Comorbidity Index, mean (SD)	0.2 (0.5)	0.4 (0.7)	0.2 (0.7)	0.5 (1.0)	1.1 (2.2)	3.4 (2.5)	0.7 (1.5)
Diabetes, n(%)	167 (0.7)	701 (1.4)	86 (0.8)	153 (1.9)	16 (2.8)	1653 (13.5)	2776 (2.6)

Myocardial infarction, n(%)	242 (0.9)	1239 (2.5)	135 (1.2)	248 (3.1)	14 (2.5)	1646 (13.4)	3524 (3.3)
Congestive heart failure, n(%)	121 (0.5)	813 (1.7)	125 (1.1)	184 (2.3)	23 (4.1)	1794 (14.6)	3060 (2.9)
Peripheral vascular disease, n(%)	190 (0.7)	1206 (2.5)	128 (1.2)	234 (2.9)	19 (3.4)	1898 (15.5)	3675 (3.5)
Cancer, n(%)	839 (3.3)	3401 (6.9)	629 (5.7)	720 (9.0)	59 (10.4)	5364 (43.7)	11012 (10.3)
Metastases, n(%)	<3	0 (0.0)	<3	>11	32 (5.6)	897 (7.3)	951 (0.9)
Kidney disease, n(%)	5 (0.0)	0 (0.0)	<3	>33	16 (2.8)	1472 (12.0)	1531 (1.4)
Leukaemia, n(%)	69 (0.3)	150 (0.3)	45 (0.4)	75 (0.9)	12 (2.1)	488 (4.0)	839 (0.8)
Lymphoma, n(%)	86 (0.3)	156 (0.3)	44 (0.4)	50 (0.6)	6 (1.1)	553 (4.5)	895 (0.8)
**Smoking ever, n(%)**	2224 (8.7)	6607 (13.5)	1491 (13.5)	1056 (13.2)	94 (16.6)	3677 (30.0)	15149 (14.2)
**Alcohol abuse, n(%)**	831 (3.2)	1793 (3.7)	439 (4.0)	271 (3.4)	22 (3.9)	903 (7.4)	4259 (4.0)
