# Supplementary material for: Machine-learning-based Phenomapping of Patients with Keratinocyte Carcinoma: Data-driven Subgrouping by Disease Burden, Comorbidities and Socioeconomic Status
Source: Acta Derm Venereol. 2026 Aug 13;106:0843. doi: 10.2340/actadv.v106.adv-2026-0843 (PMC13470789; doi:10.2340/actadv.v106.adv-2026-0843)

Figure S1: Showing the loss with respect to the number of clusters.

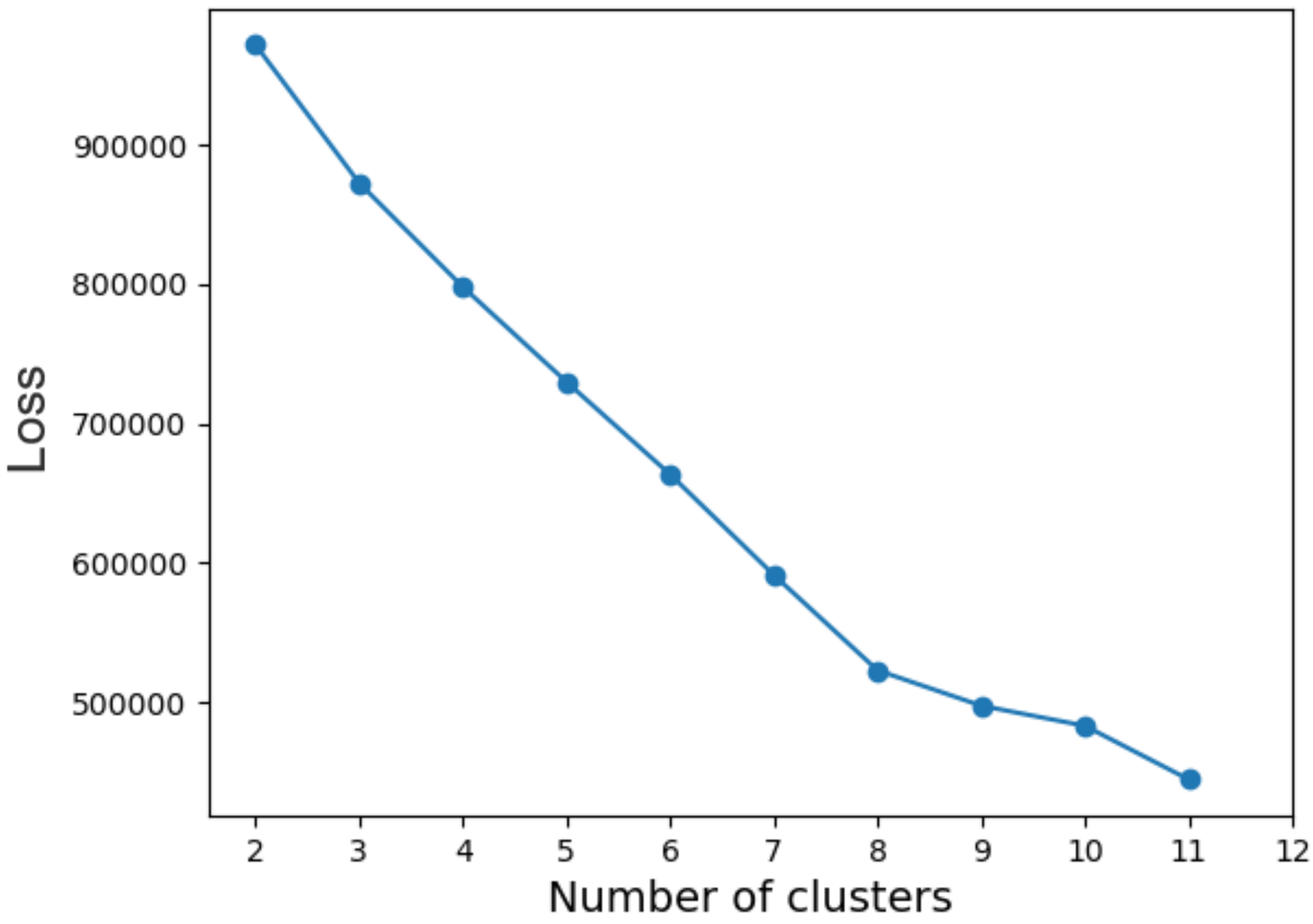

Figure S2: Showing the silhouette score with respect to the number of clusters estimated from a 50,000 random sample of the population.

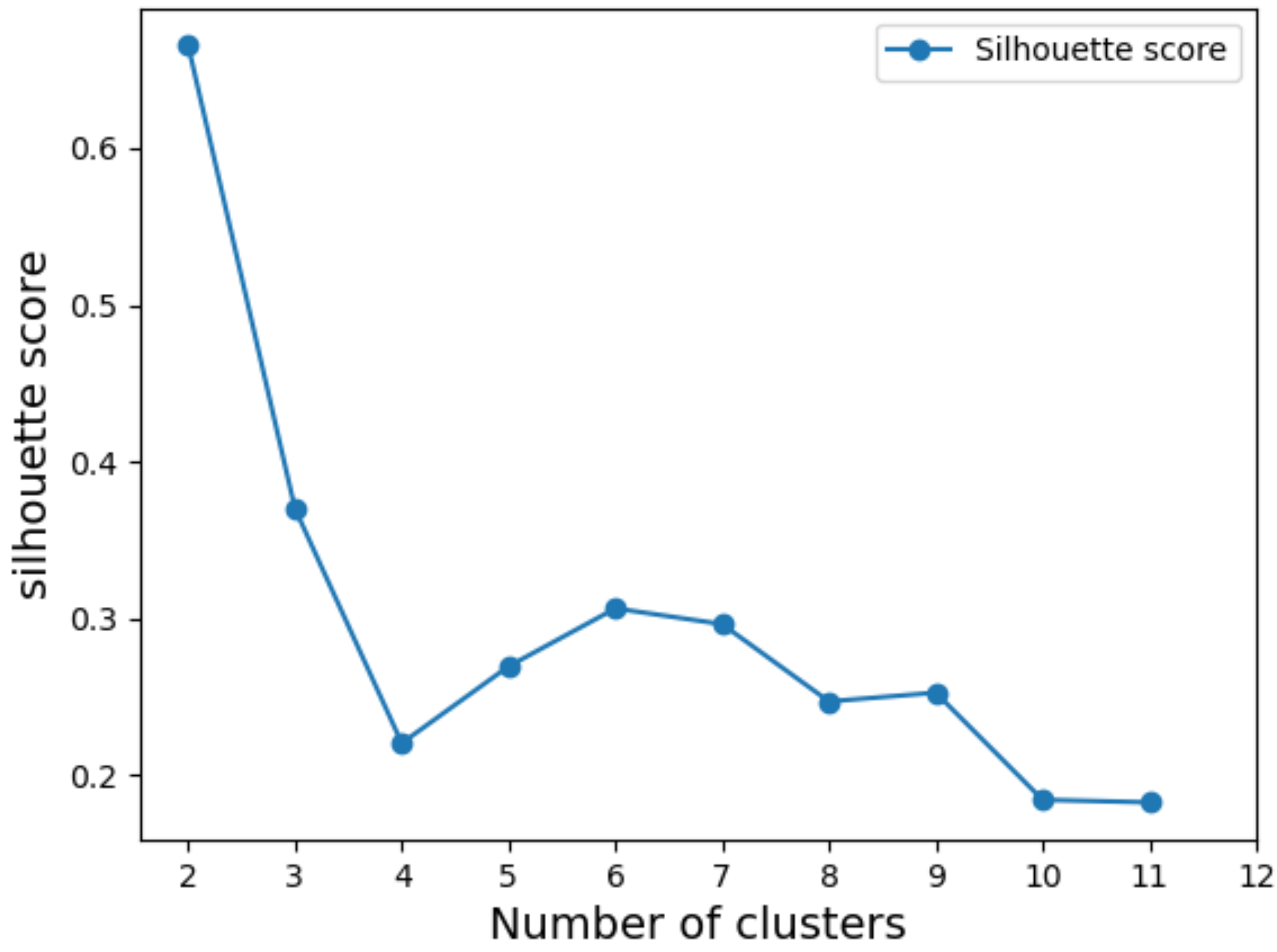

Figure S3: Showing the cluster stability with respect to the number of clusters using adjusted rand index as cluster stability score.

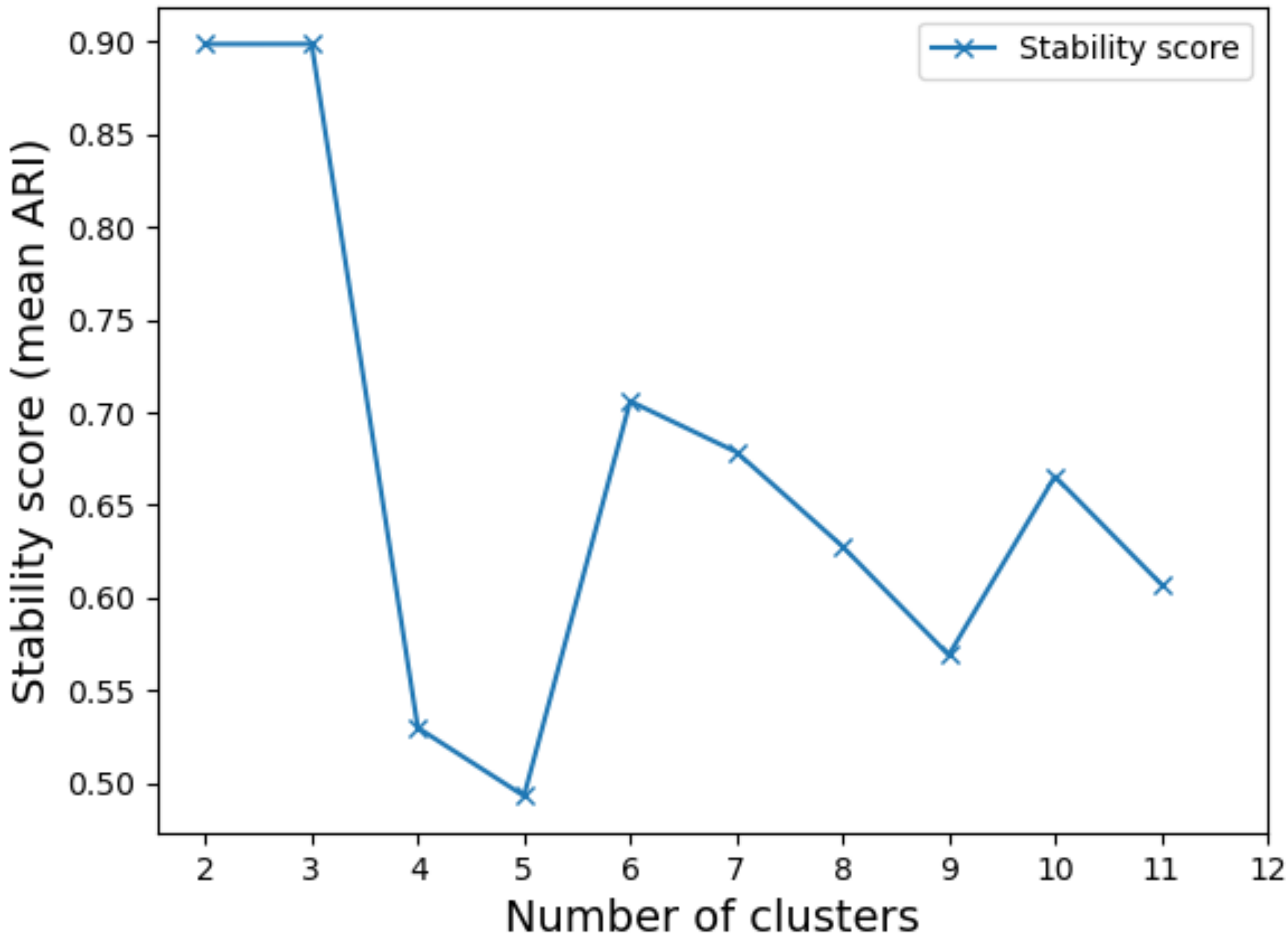

Figure S4: Histogram over percentage of different comorbidities in each cluster for the 6-cluster solution.

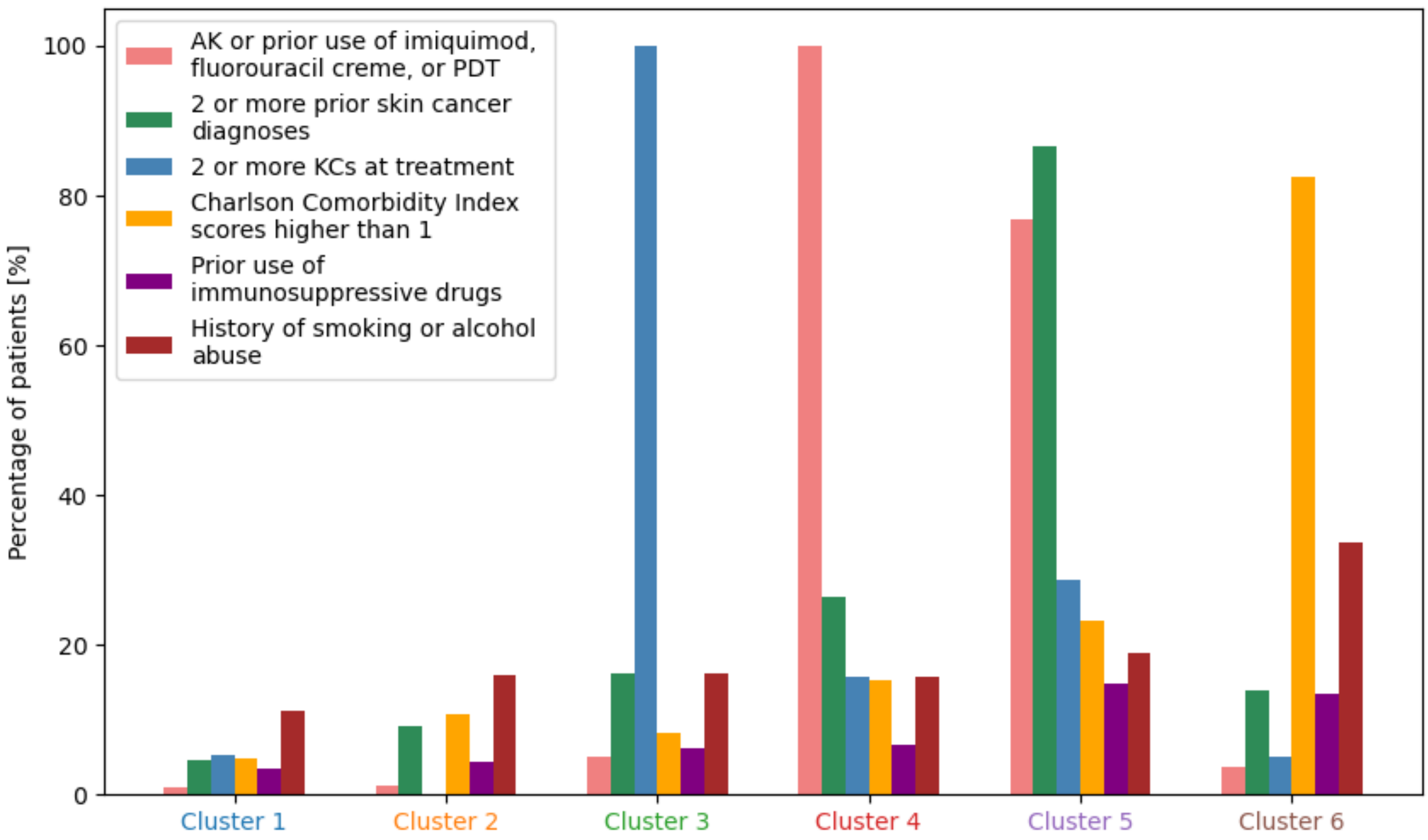

Figure S5: Heatmap over percentage point difference between each cluster compared with the rest of the KC population for the 6-cluster solution.

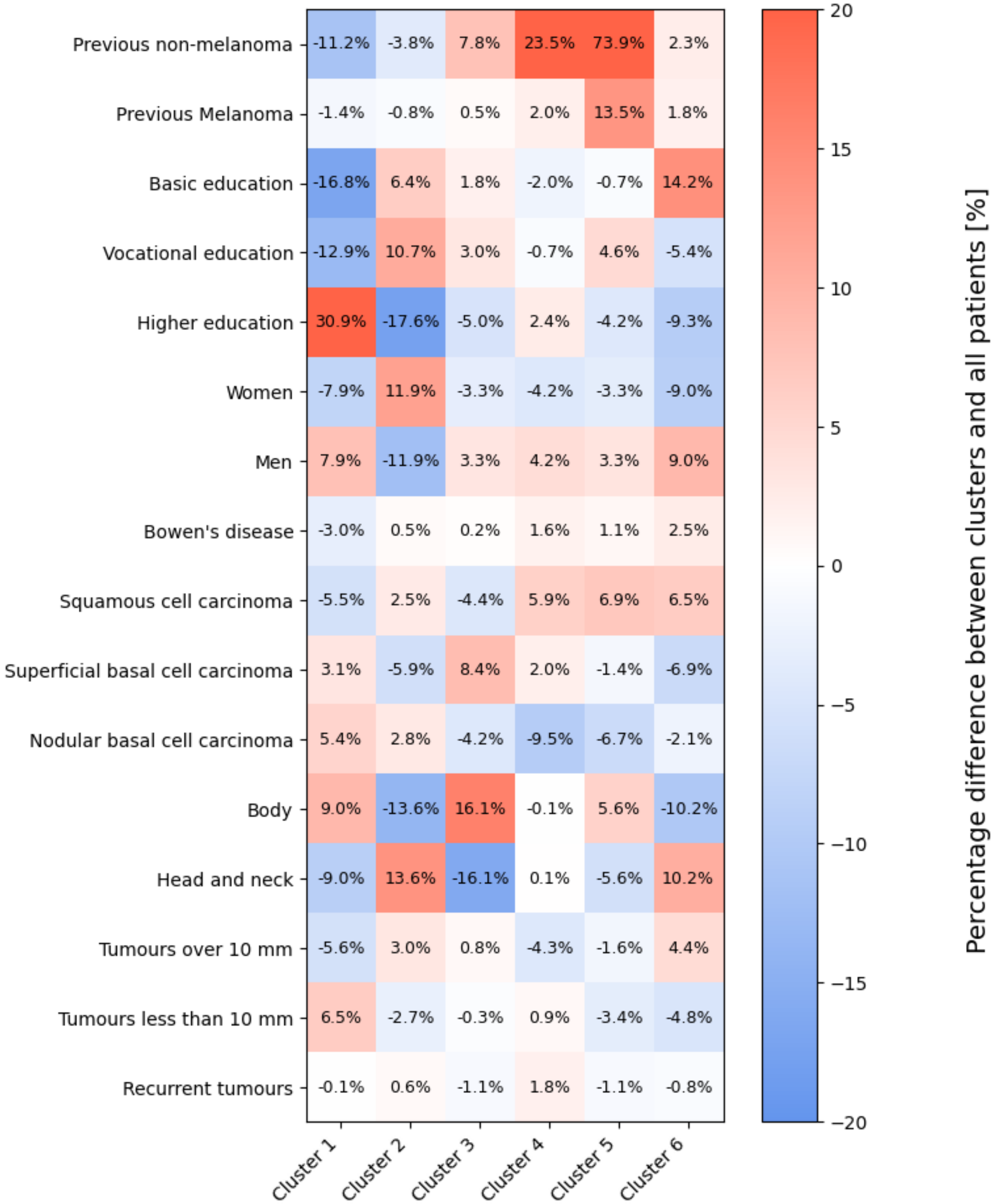

Supplement: Supplementary file 3 [file ActaDv-106-0843-s0003.pdf]
